# Experience of the management of coronary artery bypass graft only on moderate ischemic mitral regurgitation: A single-center retrospective study

**DOI:** 10.1097/MD.0000000000014969

**Published:** 2019-04-26

**Authors:** Weitie Wang, Bo Li, Yong Wang, Hulin Piao, Zhicheng Zhu, Rihao Xu, Dan Li, Kexiang Liu

**Affiliations:** Department of Cardiovascular Surgery, the second Hospital of Jilin University, Changchun, Jilin, China.

**Keywords:** coronary artery bypass, echocardiography, ischemic heart disease, mitral regurgitation

## Abstract

To summary the impact of off-pump coronary artery bypass grafting (CABG) only on patients with moderate ischemic mitral regurgitation and survival.

We retrospectively analyzed 109 patients with coronary artery disease (CAD) complicated by moderate mitral regurgitation, from January, 2008 to December, 2014, in the Department of Cardiovascular Surgery at the No. 2 Hospital of Jilin University undergoing off pump CABG only. Preoperative clinical characteristics, complications after surgery, and outcome (survivor or death) were assessed. We observed the degree of mitral valve regurgitation, left ventricular ejection fraction (LVEF), left ventricular and left atrial size, left ventricular end-diastolic volume (LVEDV) preoperative, and New York Heart Association (NYHA) functional class, postoperative 10 days before discharge, and 6 months and longer after surgery. The statistical data were processed by SPSS 19 software with computer; statistical significant difference with *P* < .05.

Overall in-hospital mortality was 2.75% (3 patients). Patients had lower mean LVEF in the postoperative compared with the preoperative period, but all the patients had higher LVEF since 6 months than preoperative period (*P* < .001). Compared with the preoperative dates, postoperative valvular regurgitation, left ventricular and atrial size and LVEDV postoperative 10 days before discharge, 6 months and more longer after surgery reduced significantly (*P* < .001). Rapid atrial fibrillation occurred in 19 cases during perioperative and returned to normal before discharge. The symptom of angina was disappeared in all patients before discharge. The mean follow-up time was 60.16 ± 17.98 months (range 36–96 months). Two patients died of major adverse cardiac events including heart failure and ventricular fibrillation. Three patients died of lung cancer, and 2 patients died of stroke during the longer follow-up.

Off-pump CABG can be performed safely in patients with CAD complicated by moderate mitral regurgitation. The efficacy of CABG only is well demonstrated by the significant improvement of LVEF and NYHA functional class, and by the decrease of left ventricular and atrial size, LVEDV, and mitral regurgitation grade.

## 1 Introduction

Mitral regurgitation (MR) is a common complication (10%) after myocardial infarction (MI).^[1]^ It is a result of the enlargement of the left ventricle, which displaces the papillary muscles and changes the proper length of chordae tendineae. The leaflet structure and subvalvular apparatus are normal, so it is a functional MR. The proper management of the coronary heart disease patients with moderate MR remains a source of controversy. In consideration of late symptoms and long-term survival, some authors advocate mitral valve repair (MVR) at the time of coronary artery bypass grafting (CABG).^[2]^ However, those holding a conservative approach believe that ischemia mitral regurgitation (IMR) will be improved after revascularizing, and CABG only can lower operative mortality.^[3–5]^ This study aimed to analyze the mid-term results of coronary artery disease (CAD) patients with moderate MR who underwent off-pump CABG only.

## 2 Methods

Retrospective analysis of patients from January, 2008 to December, 2014 was done, and 109 patients with CAD associated with moderate MR were referred to our cardiovascular surgery center for CABG. This study was approved by ethical committee rules of Jilin university (Certificate No: E2014033L), Chang Chun, China.

All the patients were diagnosed with CAD by digital subtract coronary artery angiography. Significant lesions were defined as single coronary artery luminal stenosis ≥70% or left main coronary artery (LMCA) ≥50%. The MR was caused as a consequence of the CAD and was diagnosed by Doppler transthoracic echocardiography (Philips iE Elite). The echocardiogram was analyzed by 2 experienced cardiologists who were specialized in cardiac ultrasonography. MR due to rheumatic, infectious, congenital, and degenerative diseases was excluded. Papillary muscle rupture or elongated chordae tendineae were also not considered for the study.

We obtained the clinical data as follows: age, sex, arterial blood pressure, arterial hypertension, dyslipidemia, diabetes, smoking, New York Heart Association (NYHA) functional class, outcome (survivor or death), time of stay in intensive care unit (ICU) and hospital, atrial fibrillation (AF), major procedure-related complications: respiratory complications, neurologic complications (stroke or transient ischemic attack), low cardiac output before or after surgery.

Echocardiographic findings such as left ventricular ejection fraction (LVEF), left ventricular end-diastolic volume (LVEDV), grade of MR, coronary angiography findings, and CABG results have also been recorded. MR is strictly quantified according to European Society of Echocardiography diagnostic criteria.^[6]^

Graft patency was assessed by graft angiography or coronary artery computed tomography (CT) scan, and each graft was viewed in at least 2 orthogonal planes and graded A (excellent), B (fair), or O (occluded) by separate assessment of proximal and distal anastomoses and bypass trunks; grades A and B was designated as patency.

**Figure 1:**
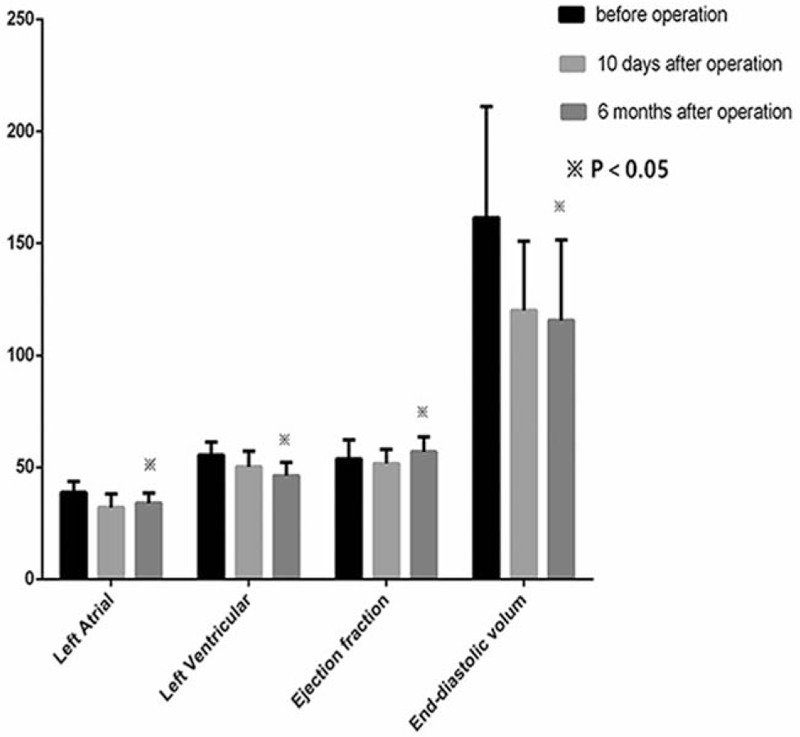
Postoperative echocardiography data in the coronary artery bypass grafting only during follow-up. ^∗^*P* < .001: compare with before operation.

All the data were performed with SPSS 19 on computer and were expressed as mean ± standard deviation. SNK-q test was used for multiple comparisons among groups. Chi-square test was used for enumeration data analysis. Values of *P* < .05 were considered statistically significant (Fig. 1).

### 2.1 Ethics approval and consent to participate

The study design was approved from ethical committee rules of Jilin University, Chang Chun, China. Written informed consent was obtained from the patient for publication of this research article.

## 3 Results

Among 109 patients, 76 (69.72%) men underwent off-pump CABG only. The mean age was 63.67 ± 6.89 years (range 45–76 years). Clinical characteristics and angiographic data are presented in Table 1.

**Table 1:**
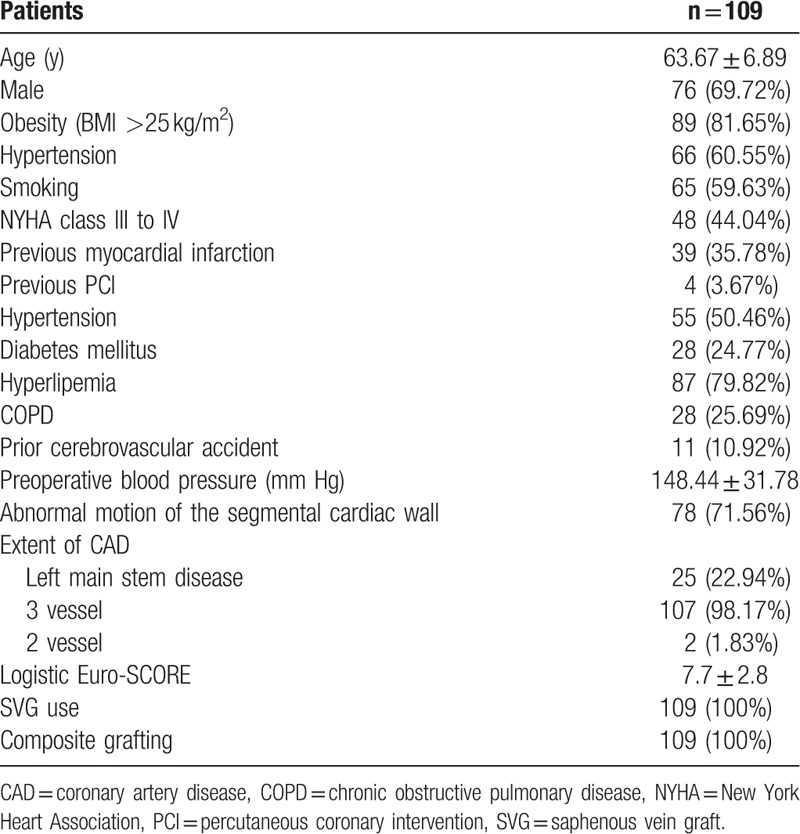
Baseline and procedural characteristics.

### 3.1 Early mortality and outcome

The total mortality rate was 2.75% (3 patients) in hospital. Two (1.80%) patients died of low cardiac output on the second day after off-pump CABG, and 1 (0.95%) patient died of multiorgan failure. All patients got re-examined by graft angiography or coronary artery CT scan at the first year after discharge (Table 2).

**Table 2:**
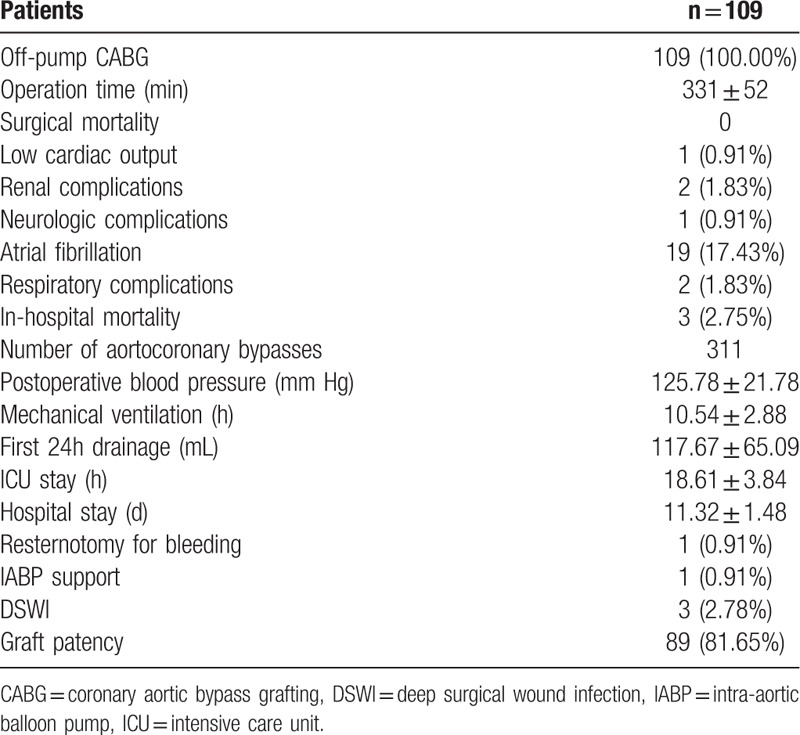
Intraoperative and postoperative patient's data.

### 3.2 Late mortality and outcome

Follow-up after discharge was completed in all survivors, with a mean duration of 60.16 ± 17.98 months (range 36–96 months). All patients received echocardiography, and 23 patients received graft angiography or coronary artery CT scan. Two patients died of major adverse cardiac events (MACE) including heart failure and ventricular fibrillation. Three patients died of lung cancer, and 2 patients died of stroke during the longer follow-up (Table 3).

**Table 3:**
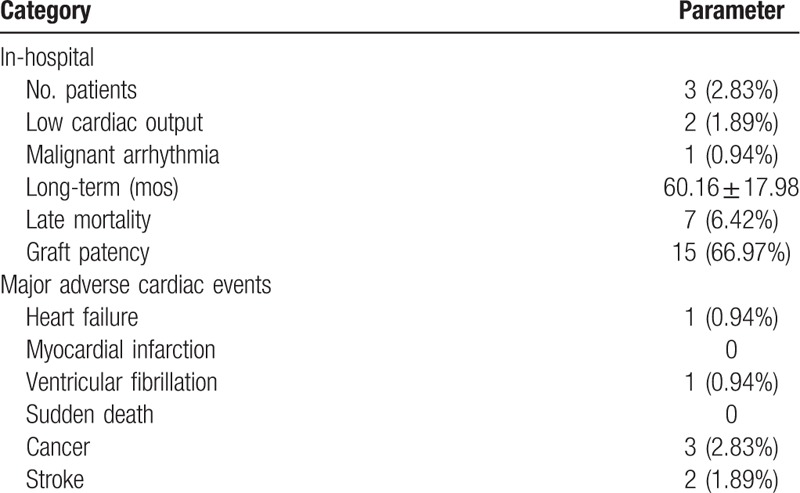
Causes of death.

### 3.3 Effect on postoperative MR

Among all patients who had mild-to-moderate or moderate, or moderate-to-severe MR, respectively, 52 (49.06%), 50 (47.17%), and 4 (3.77%) preoperation. The latest follow-up showed that trivial MR was present in 75 (70.75%) patients, mild MR was present in 29 (27.36%) patients, and moderate MR was present in 2 (1.89%) patients (χ^2^ = 129.84, *P* < .001), which suggest that off-pump CABG alone had an effect on improving the degree of regurgitation of the patients with moderate ischemic mitral.

### 3.4 Echocardiographic results

All patients were suggested re-examine transthoracic echocardiography (TTE) at the first 6 months and every year after discharge. MR grade was improved in 104 patients (98.11%). Statistically significant difference in LV reversal remodeling was observed in LV (*P* < .001) and LVEDV (*P* < .001) 10 days after operation comparing with preoperation. The left atrial (LA) size changed from 38.86 ± 4.85 to 32.08 ± 5.99 (*P* < .001). MR changed from 3.12 ± 0.33 to 0.62 ± 0.49 (*P* < .001). LVEF presented lower in the postoperative period (10 days) than in the preoperative period, but improved significantly after 6 months compared with preoperative period, showing an improvement in LV function. Mean NYHA class showed improvement from 3.03 ± 0.63 to 2.21 ± 0.84 (*P* < .001).

During the longer follow-up, data showed an improvement in MR compared with preoperation (*P* < .001) and 10 days after operation (*P* < .001). Degree of MR increased than 10 days after operation as time goes on but still in trace. The mean LVEF, NYHA class improved and LV reduce significantly (*P* < .001) as time went on during the follow-up compared with 10 days after operation. LVEDV reduced significant difference compared with preoperation (*P* < .001), but had no significant difference compared with 10 days after operation (*P* = .34) (Table 4).

**Table 4:**
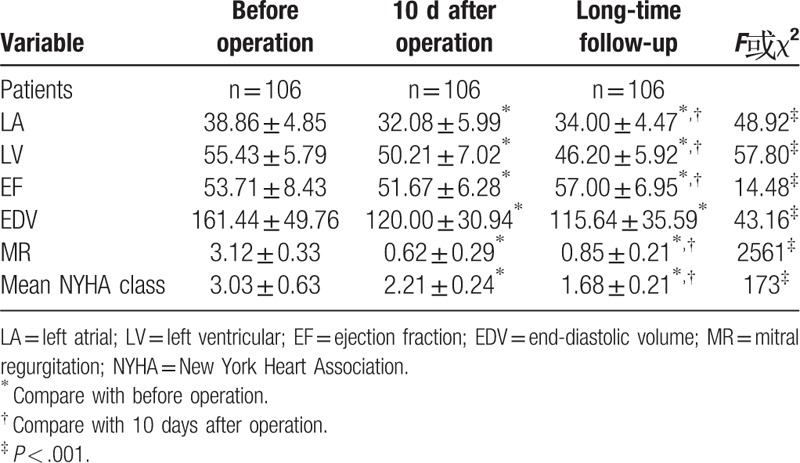
Echocardiography data.

## 4 Discussion

Chronic IMR is a functional disease which presents in 30% to 50% of patients with a MI.^[7]^ LV geometric remodeling displaces the papillary muscles and then influences the chordae tendineae resulting in valvular incompetence. When assessing MR by echocardiography, the echocardiographer should: confirm underlying chronic ischemic heart disease; exclude intrinsic pathology in the chordae and leaflets; establish IMR by assessing papillary muscles displacement and LV.^[8]^ TTE is regularly used to evaluate the grade of MR according to severity classification: absent (0+), trace (1+), mild (2+), moderate (3+), or severe (4+) of MR. For 1+ MR or less and for 4+MR, most authors have reached an agreement with the management.^[9]^ However, proper surgical statics still remains controversial for the range of 2+ to 3+ MR. In this study, we aim to evaluate the effect of CAD patients with moderate MR undergoing off-pump CABG only and its effect on outcomes in terms of the size of LA and LV, LVEDV, LVEF, postoperative NYHA functional class, and longer survival.

People suggest adding MVR to CABG considers that MVR eliminates the volume overload and the loss of volume ejected toward the LA displacing the entire cardiac output in the correct direction (to the aorta). Because of its effectiveness and reliability, there are many studies aimed to evaluate the effects and prognosis of adding MVR to CABG and CABG alone in patients with MR; CABG-combining MVR procedures show low mortality, and better follow-up^[10–12]^ significantly improves survival rate. The residual or recurrent rate of moderate MR at 2-year follow-up in CABG-only group is more susceptible (32.3% vs 11.2% for the CABG and MV repair group; *P* < .001).^[10]^ In addition, combined CABG and MVR can effectively reduce the vena contracta width (−3.40 ± 0.2 mm) than CABG only (−1.45 ± 0.7 mm; *P* = .02).^[11]^

Those believe CABG only can produce effective improvement on LVEF, MR, and NYHA so as to achieve good long-term survival.^[13]^ They think that this conservative approach can restore the perfusion, and then recover the function and reduce the dimension of the left ventricle, finally leading to a significant reduction in MR. Kim et al's^[14]^ study shows that a similar 5-year survival between CABG combining MVR and CABG alone (44% ± 5% vs 41% ± 7%; *P* = .53). In addition, several studies including a 2009 meta-analysis^[15]^ have reported no survival benefit to adding MV repair to CABG (95% confidence interval [CI] 0.90–1.14, *P* = .73) for patients with IMR. Byung et al^[6]^ reported that during a median follow-up of 78.0 months, the incidence of all-cause mortality was 232 (5.8% per year) in CABG-only group and 54 (7.5% per year) in CABG + MV surgery group (*P* = .12), which suggests that a concomitant MV surgery seems to confer no significant clinical benefits. This study also points that CABG plus MVR group does have a significantly increased rate of neurological events (14 events) than CABG-only group (4 events; *P* = .02).^[16]^ In addition, Sun et al^[17]^ demonstrate that outcomes of patients with moderate MR are relevant with LVEF and timing after infarction. So, in patients with good LVEF and early timing after infarction, CABG only will get excellent results.

The effect of CABG only on CAD patients with moderate IMR is still unclear. To date, there are few studies to investigate this issue. Thus, we aim to analyze whether CABG only can improve MR grade, LV function, and NYHA and long-time survival rate, which can provide clinical evidence for the future treatment of these patients. In our retrospective analysis, compared with preoperation, MR grade, size of LA and LV, LVEDV decreasing significantly after 10 days, 6 months and longer after CABG only. Furthermore, the in-hospital mortality, postoperative mean NYHA class, and long-time survival show no significant difference with other studies.

Overall, surgical revascularization results in significant improvements in ventricular function, as measured by means of the LVEDV and LVEF. In our study, CABG alone is able to reduce MR grade in 104 (98.11%) patients, whereas there are 2 moderate MR remaining stable after operation and longer follow-up. We found that these 2 people have a history of old MI. The perfusion of these scarce areas cannot be restored after CABG, which make LV reversal remodeling unpredictable.

During longer follow-up, the 2 patients with moderate MR do not translate into a higher risk of death, whereas 2 deaths of MACE are patients with trace MR. Few additional deaths occur during follow-up, which is consistent with results that have been published previously.^[13–17]^ In addition, we observed that the 2 patients with moderate MR have higher rates of MACE like hospital readmission during heart failure.

We conclude the good result for: excellent echocardiographic assessment not only of ischemic IMR and MR severity, but also confirm sequelae of ischemic heart disease such myocardial scarring, wall thinning, and wall motion abnormalities; all the patients’ blood pressure after CABG and discharge were controlled within 120 to 140 mm Hg, which we think the severity of MR (and also LVEF) is strongly influenced by cardiac afterload.

## 5 Conclusions

To conclude, our management shows patients with moderate regurgitation are more likely to benefit from CABG only, and shows no significant differences with other studies. Although the published studies show a large degree of heterogeneity and tend to favor the combined CABG plus MVR procedure in CAD patients with IMR, our study shows these patients have benefited from CABG surgery, which makes a contribution for future larger-scale trials.

### 5.1 Limitation

The current trial has several limitations. Firstly, this study was still a retrospective observational study with a single central which may influence the generalizability. So a final determination would need a prospective, multicenter study with larger sample size. Secondly, myocardial viability was not used to evaluate preoperative and effectiveness of revascularization after CABG. Thirdly, this essay has no control group because CABG plus mitral valve repair was carried in recent years in our department for only 23 cases. So larger sample size will be added as a control group in the future.

## Author contributions

WWT, XRH, and LKX conceived of the study, and participated in its design and coordination and helped to draft the manuscript. WWT and DL participated in the design of the study and drafted the manuscript. HLP, YW, BL, and ZCZ carried out the data collection and statistical analysis. All authors read and approved the final manuscript.

**Conceptualization:** Weitie Wang.

**Data curation:** Weitie Wang, Hulin Piao.

**Formal analysis:** Weitie Wang, Hulin Piao.

**Funding acquisition:** Dan Li.

**Investigation:** Yong Wang.

**Methodology:** Yong Wang, Zhicheng Zhu, Kexiang Liu.

**Project administration:** Zhicheng Zhu, Rihao Xu, Dan Li.

**Resources:** Bo Li, Rihao Xu.

**Software:** Bo Li, Zhicheng Zhu, Rihao Xu, Dan Li.

**Supervision:** Dan Li, Kexiang Liu.

**Validation:** Dan Li, Kexiang Liu.

**Visualization:** Kexiang Liu.
